# Mechanisms of Lysophosphatidic Acid-Mediated Lymphangiogenesis in Prostate Cancer

**DOI:** 10.3390/cancers10110413

**Published:** 2018-10-31

**Authors:** Pei-Yi Wu, Yueh-Chien Lin, Yuan-Li Huang, Wei-Min Chen, Chien-Chin Chen, Hsinyu Lee

**Affiliations:** 1 Institute of Cellular and Organismic Biology, Academia Sinica, Taipei 11529, Taiwan; d96b41003@ntu.edu.tw; 2 Department of Life Sciences, National Taiwan University, Taipei 10617, Taiwan; d01b41001@ntu.edu.tw (Y.-C.L.); f03b21010@ntu.edu.tw (W.-M.C.); 3 Department of Biotechnology, Asia University, Taichung 41354, Taiwan; yuanli@asia.edu.tw; 4 Department of Medical Research, China Medical University Hospital, China Medical University, Taichung 40402, Taiwan; 5 Department of Pathology, Ditmanson Medical Foundation Chia-Yi Christian Hospital, Chiayi 60002, Taiwan; 6 Department of Cosmetic Science, Chia Nan University of Pharmacy and Science, Tainan 71710, Taiwan; 7 Department of Electrical Engineering, National Taiwan University, Taipei 10617, Taiwan; 8 Institute of Biomedical Electronics and Bioinformatics, National Taiwan University, Taipei 10617, Taiwan; 9 Center for Biotechnology, National Taiwan University, Taipei 10617, Taiwan

**Keywords:** LPA, LPA receptor, prostate cancer, VEGF-C, lymphangiogenesis

## Abstract

Prostate cancer (PCa) is the most common noncutaneous cancer in men worldwide. One of its major treatments is androgen deprivation therapy, but PCa frequently relapses as aggressive castration resistant local tumors and distal metastases. Hence, the development of novel agents or treatment modalities for advanced PCa is crucial. Many tumors, including PCa, first metastasize to regional lymph nodes via lymphatic vessels. Recent findings demonstrate that the bioactive lipid lysophosphatidic acid (LPA) promotes PCa progression by regulating vascular endothelial growth factor-C (VEGF-C), a critical mediator of tumor lymphangiogenesis and lymphatic metastasis. Many of the underlying molecular mechanisms of the LPA–VEGF-C axis have been described, revealing potential biomarkers and therapeutic targets that may aid in the diagnosis and treatment of advanced PCa. Herein, we review the literature that illustrates a functional role for LPA signaling in PCa progression. These discoveries may be especially applicable to anti-lymphangiogenic strategies for the prevention and therapy of metastatic PCa.

## 1. Introduction

Prostate cancer (PCa) is one of the most common cancers in aging men. According to the statistics based on 2011–2015 cases from the National Cancer Institute, USA, the number of new cases of PCa was 112.6 per 100,000 men per year, and the number of deaths was 19.5 per 100,000 men per year during this period. Approximately 11.2% of men will be diagnosed with PCa at some point during their lifetime. The 5-year survival rate for localized and regional PCa is 100%, whereas the rate for distant PCa decreases to 30%. PCa tumors usually originate as prostatic adenocarcinomas, a term that describes uncontrolled and abnormal growth in the glandular tissue of the prostate. The male hormone, androgen, is known to be a major regulator of PCa growth and survival, which acts by both stimulating proliferation and inhibiting apoptosis [1,2,3,4]. Therefore, hormone therapy, which is also called androgen deprivation or androgen ablation, has become a standard clinical treatment for PCa. Although hormone therapy can suppress the progression of PCa [2,3], the cancer frequently relapses as a more malignant form. Upon PCa relapse, tumors frequently contain androgen-refractory cells (known as castration-resistant prostate cancer, CRPC), which are characterized as more aggressive and highly metastatic compared to androgen-dependent cells [5,6]. Therefore, current clinical therapeutic strategies of such advanced PCa are chemotherapy, radiation therapy, and immunotherapy. Meanwhile, new therapeutics for patients with advanced PCa are urgently needed.

Lysophosphatidic acid (LPA) is an extraordinary bioactive lipid species, which is primarily derived from membrane phospholipids. The binding of LPA to a family of G protein-coupled receptors (GPCRs), termed LPA receptors (LPA_1–6_), activates different G-protein α subunits (G_s_, G_i_, G_q/11_, G_12/13_) to initiate cellular effects [7,8]. Through this mechanism, LPA functions as a pathological mediator in various types of cancers, mainly functioning as a regulator of cancer progression [9,10]. In PCa, LPA binding stimulates signaling cascades that promote cell proliferation, migration, invasion, and survival [11,12,13,14,15]. Notably, LPA has been shown to enhance the growth and metastasis of several cancers by increasing vascular endothelial growth factor-A and -C (VEGF-A and VEGF-C) expression and secretion, which stimulates angiogenesis and lymphangiogenesis [16,17,18]. These effects of LPA on VEGF-A and VEGF-C have been observed in PCa cells, where VEGF-C (but not VEGF-D) was found to be especially important in mediating lymphangiogenesis and tumor metastasis in advanced PCa [18,19,20]. Thus, it has become clear that LPA and its receptors are critical factors in PCa progression and metastasis. Herein, we review the molecular regulation of LPA-mediated lymphangiogenesis in PCa.

## 2. Lymphangiogenesis and Metastasis in PCa

**Figure 1:**
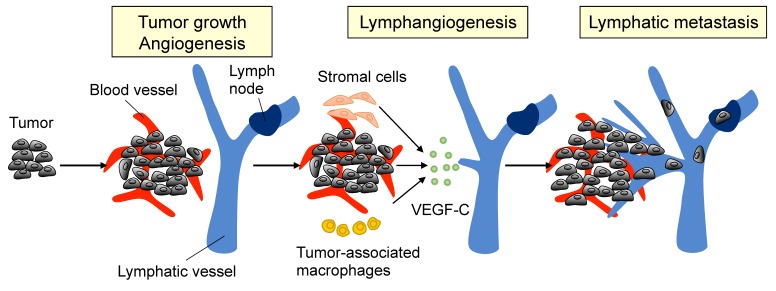
Tumor lymphangiogenesis. The primary tumor, as well as stromal cells and tumor-associated macrophages, release vascular endothelial growth factor-C (VEGF-C) to initiate tumor lymphangiogenesis. Once VEGF-C activates lymphatic endothelial cells (LECs) by binding to VEGFR-3, the cells proliferate and migrate to form new lymphatic vessels in the primary tumor.

The term lymphangiogenesis describes the formation of new lymphatic vessels from the pre-existing vasculature (Figure 1). This critical process is widely known to influence metabolism, immunity, and disease pathology, including cancer metastasis. In particular, advanced PCa is aggressive and highly metastatic, involving the loss of cell adhesion, the enhancement of local invasion, angiogenesis, and lymphangiogenesis [21]. Lymphangiogenesis is known to be induced by vascular endothelial growth factors (VEGFs) [22], of which there are six, including VEGF-A, -B, -C, -D, -E, and placental growth factor (PIGF). Each VEGF binds to the VEGF tyrosine kinase receptors (VEGFRs): VEGFR-1, VEGFR-2, and VEGFR-3. It has been reported that VEGF-C is a major lymphangiogenic regulator that activates lymphangiogenesis-associated signaling pathways by binding to VEGFR-3 [23]. In PCa, clinical evidence has suggested that paracrine activation of lymphatic endothelial VEGFR-3 by tumor-secreted VEGF-C might be involved in lymphatic metastasis [24,25]. This idea is further supported by in vivo animal experiments, which showed that overexpression of VEGF-C in PCa cells increases metastasis to lymph nodes and the lungs [26]. Therefore, researchers wondered whether the blockade of VEGFR-3 signaling might also be able to suppress advanced PCa. Indeed, Burton et al. [27] provided evidence that the treatment of PCa tumor-bearing mice with a VEGFR-3 antagonist can successfully inhibit lymphangiogenesis and cancer metastasis. Interestingly, the reduction of androgens in cultured PCa cells upregulates the level of VEGF-C [28] through the production of reactive oxygen species (ROS) and the activation of the small GTPase, RalA [29]. This observation may explain why conventional androgen deprivation therapy in clinical patients often leads to the relapse of aggressive PCa. Furthermore, the observations suggest that blocking the VEGF-C/VEGFR-3 axis in PCa might be better than hormone therapy as a treatment for advanced PCa.

## 3. Lysophosphatidic Acid Signaling Pathway

LPA consists of a polar phosphate head group, a glycerol backbone, and a long hydrophobic fatty acyl group, and was first identified as a vasodepressor in incubated feline plasma in 1979 [30,31]. This lipid growth factor is present in high levels in the serum [32], saliva [33], and ascites from cancer patients [9]. Activated platelets also serve as a major source of LPA [34,35,36]. After decades of investigations, LPA was found to be mainly synthesized from various membrane phospholipids, through either intracellular or extracellular mechanisms. The intracellular biosynthesis of LPA is catalyzed by three major enzymes in the endoplasmic reticulum (ER) and mitochondrial membrane: glycerophosphate acyltransferase (GPAT), phospholipase A (PLA), and acylglycerol kinase (AGK) [37,38,39]. Remarkably, excessive AGK expression was found in PCa patients and LNCaP cells [40]. The overexpression of AGK in PCa cells enhances both cell proliferation and migration [38], suggesting intracellular synthesized LPA has as yet undescribed roles in determining malignancy-related characteristics of PCa cells. Meanwhile, LPA is also synthesized extracellularly by a secreted enzyme, autotaxin (ATX) [41]. ATX has lysophospholipase D (lysoPLD) activity that converts lysophosphatidylcholine (LPC) to LPA [42,43,44], and clinical evidence indicates that the concentration of ATX in human serum samples is strongly correlated with the plasma LPA concentration [45]. Furthermore, LPA levels in the serum of *ATX^+/−^* mice were reported to be decreased by half, supporting a role for ATX as the dominant synthetic enzyme for the generation of extracellular circulating LPA. Similar to the production of LPA in ovarian cancer cells [46], PCa cells also release and respond to LPA as an autocrine signal [47]; this signaling is enhanced by the increased expression of ATX in PCa [38]. Sequencing data obtained from PCa patients (cBioPortal) has shown the strong clinical significance of ATX. For example, about 6% prostate adenoma patients demonstrated amplification of ATX in the TCGA dataset (330 patients) [48] and 5% in the MSKCC/DFCI dataset (1013 patients) [49], whereas the rate in metastatic PCa patients increased to 12% in the SU2C/PCF dataset (150 patients) [50] and 20% in the MCTP dataset (61 patients) [51]. Interestingly, neuroendocrine PCa showed the highest rate of ATX amplification to 38% in the Trento/Cornell/Broad dataset (114 patients) [52]. The protein level of ATX in PCa patients was also shown to be higher in PCa cells than in non-neoplastic prostate cells [20,40]. Moreover, ATX gene expression was shown to be elevated in androgen-insensitive PCa, PC-3, and DU-145 cells relative to androgen-dependent LNCaP cells [53], implying that advanced PCa cells might also exhibit LPA-initiated signaling and cellular functions. Interestingly, the ATX concentration was decreased in the serum of postoperative patients with PCa [54], supporting the idea that ATX acts as an autocrine growth factor in human PCa. Together, these studies suggest that the ATX–LPA axis might be a crucial indicator of PCa progression.

**Figure 2:**
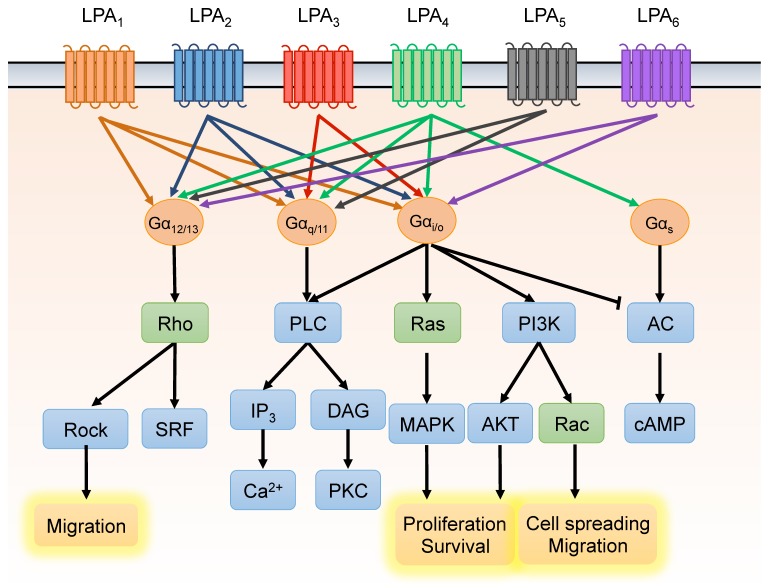
Lysophosphatidic acid (LPA) receptors and downstream signaling pathways. LPA binds LPA receptors (LPA_1–6_) with varying affinities, triggering various downstream signaling cascades via receptor coupling to four different heterotrimeric G proteins. Through this signaling mechanism, LPA mediates cellular events such as cell proliferation, apoptosis, migration, and differentiation.

LPA receptors are canonical GPCRs that reside on the plasma membrane by virtue of seven transmembrane domains. Upon binding to extracellular LPA, the receptors are activated and subsequently evoke downstream cellular signaling cascades with various pathophysiological functions in almost all types of mammalian cells [55]. At least six types of LPA receptors have been identified and are classified in two groups; LPA_1–3_ are closely related and were initially identified as the endothelial differentiation gene (EDG), while LPA_4–6_ belong to the P2Y purinergic receptor family. LPA receptors are coupled to trimeric G proteins, including G_s_, G_i/o_, G_q/11_, and G_12/13_, which exert pathophysiological functions via small GTPase, Ras, Rho, and Rac, modulation of a variety of signaling cascades [7,8] (Figure 2). The expression profiles of LPA receptors are incredibly diverse and depend on the cell type. In PCa, abundant expressions of LPA_1_, LPA_3_, and LPA_6_ were reported [56,57,58]. Additionally, the levels of LPA receptors differ between androgen-dependent and androgen-insensitive PCa cells [56]. In androgen-dependent LNCaP cells, LPA_3_ is the LPA receptor with the highest expression level. On the other hand, in androgen-insensitive PCa, PC-3, and DU-145 cells, high levels of LPA_1_ were shown to be expressed compared to LPA_3_. Clinical datasets in cBioPortal.org also provide evidence of LPA receptor gene/mRNA expression in both primary and metastatic PCa patients. The data show that in prostate adenocarcinoma, both gene and mRNA expressions of LPA_1–5_ are upregulated and mutually exclusive, whereas LPA_6_ are mostly deleted. In metastatic PCa, gene amplification and deletion are mixed and mutually exclusive. Notably, in neuroendocrine PCa, LPA_1–5_ are massively amplified compared to the other stages of the PCa. This data indicates LPA receptors play different roles in the different stages of PCa, and LPA signaling might play a more important role in neuroendocrine PCa than in the other stages of the PCa. Stable expression of LPA_1_ in LNCaP cells positively regulates LPA-induced cell proliferation in vitro and in vivo, indicating that LPA_1_ plays a central role in transducing proliferative signals in PCa [56]. In addition, the switching of LPA receptor expression from LPA_3_ to LPA_1_ may be involved in PCa progression to androgen independence. To date, most studies have focused on the functions of LPA_1_, LPA_2_, and LPA_3_ in PCa; however, our group has also demonstrated that LPA_6_ is highly expressed in PCa [59]. The pathophysiological roles of LPA_6_ in PCa remain unclear and require further investigation.

## 4. LPA Promotes VEGF-C Expression and Lymphangiogenesis of PCa

In the past decade, our group has made a consistent effort to elucidate the essential roles of LPA signaling in lymphatic vessel development. We found that the knockdown of LPA_1_ in zebrafish embryos results in severe defects in thoracic duct development [60], suggesting that this LPA receptor is required for lymphatic endothelial cell development during embryonic lymphangiogenesis. Our results also showed that LPA promotes in vitro capillary tube formation in human umbilical vein endothelial cells (HUVECs); this process is dependent on both LPA_1_ and LPA_3_ [61,62]. Another group found comparable LPA-induced endothelial tube formation in lymphatic endothelial cells [63]. Interestingly, we found that LPA activates VEGF-C to induce expression of lymphatic markers, including Prox1 and podoplanin, in HUVECs. As such, HUVECs respond to LPA stimulation via LPA_1_ and LPA_3_, and transactivation of EGFR, eventually inducing VEGF-C expression and secretion, which in turn, activates VEGFR-3. These findings were detailed in a publication that was the first to demonstrate LPA regulation of VEGF-C expression. Since PCa has abundant VEGF-C and ATX, we then hypothesized that LPA could promote VEGF-C production in PCa cells, as was observed in primary endothelial cells. Our group provided direct evidence that LPA stimulates transcription-dependent VEGF-C production in PCa cells. The detailed mechanism involves LPA_1_ and LPA_3_, the production of ROS, and the lens epithelium-derived growth factor (LEDGF), which leads to the transcriptional regulation of VEGF-C in PC-3 cells [18]. Since ROS production has been considered a potential target for cancer therapies, we further focused on the machinery of LPA-mediated ROS generation. We found that the NADPH oxidase (Nox) pathway controls LPA-induced ROS generation [19], and Nox activation is dependent on phospholipase C (PLC) and protein kinase C (PKC) [19]. Hence, our current understanding of the LPA-induced signaling events in PCa may facilitate the design of treatment strategies to inhibit tumor lymphangiogenesis and lymphatic metastasis of PCa.

## 5. Hyperglycemia Contributes to PCa Progression by Promoting VEGF-C Expression

Several clinical studies and meta-analyses have revealed that diabetes mellitus-related hyperglycemia is positively associated with an increased risk for various types of cancers, including liver, pancreas, colorectal, kidney, bladder, endometrial and breast cancers [64,65]. Additionally, hyperglycemia was shown to increase the incidence and mortality of cancers [66], potentially by promoting the growth and metastasis of cancer cells [67,68]. In particular, studies have demonstrated that hyperglycemia promotes the progression of PCa. For example, an in vitro study indicated that hyperglycemia promotes the growth of androgen-independent DU145 cells, but a similar significant effect was not observed in androgen-dependent LNCaP cells [69], suggesting that proper control of blood glucose may suppress the growth of advanced PCa. Indeed, an antihyperglycemic agent, metformin, was utilized in the treatment of PCa and effectively decreased the prostate specific antigen (PSA) level, epithelial-mesenchymal transition (EMT), and proliferation of PCa [70,71,72].

Recently, we have begun to explore whether VEGF-C-mediated tumor lymphangiogenesis plays a role in high blood glucose promotion of advanced PCa progression. Unlike normal differentiated cells, which mostly rely on mitochondrial oxidative phosphorylation to provide ATP for cellular processes, aggressive cancer cells tend to utilize aerobic glycolysis to generate sufficient energy for active cellular proliferation; this preference is known as the Warburg effect [73]. Multiple reports have suggested a link between LPA signaling and glycolysis. In one report, LPA was found to induce glycolysis in microglial cells [74], and in another, inositol polyphosphate phosphatase 1 (INPP1), which is highly expressed in various aggressive cancer cells, was found to promote glycolysis and stimulate LPA synthase in a feed-forward mechanism [75]. Additionally, LPA was shown to drive aerobic glycolysis via increases in the GLUT glucose transporter (GLUT) and hexokinase 2 (HK2) to provide energy for biomass accumulation in tumors [75]. Furthermore, several reports have provided evidence that the LPA–Rac–Nox–ROS–HIF1α axis contributes to metabolic adaption to aerobic glycolysis in ovarian cancer [76,77,78], and these signaling mechanisms are similar to those identified in our previous studies [18,19,79].

Our recent study demonstrated that PC-3 cells respond to high glucose conditions by expressing VEGF-C [80], but LNCaP cells do not. To our surprise, we found that high glucose conditions induced VEGF-C expression via the upregulation of ATX [80]. This finding provides a potential link to our previous studies, which described the LPA induction of VEGF-C. It is, therefore, possible that elevated glucose may be an upstream signal for advanced PCa cells to induce ATX, which generates LPA and subsequently upregulates VEGF-C. Moreover, we found that high glucose also promotes the expression of calreticulin (CRT) [80], an ER chaperone which regulates intracellular calcium mobility and cellular functions, such as cell migration [81]. The effect on CRT is particularly interesting because its depletion has been shown to significantly suppress the tumor growth and lymphangiogenesis of PC-3 tumor xenografts [20], in addition to more recent findings that CRT regulates ATX and VEGF-C expression [80]. Interestingly, our observations suggest that LPA triggers a positive feedback loop, promoting CRT expression, which, in turn, stimulates LPA-induced VEGF-C production [20]. We also showed that phosphorylation of the eukaryotic initiation factor α (eIF2α), an ER stress indicator, contributes to the regulation of CRT expression via ROS production. These results suggest that CRT acts as a critical regulator of both LPA and VEGF-C production through complicated molecular machinery in PCa cells. In fact, CRT also acts as an AU-rich element (ARE) binding protein, which regulates mRNA degradation [82,83,84]. By analyzing an online ARE database, we found that the mRNAs for both ATX and VEGF-C have AREs in their 3′-untranslated region (UTR), suggesting CRT might mediate the stability of ATX and VEGF-C mRNA via direct binding. This hypothesis will be tested in future experiments. Interestingly, decreased integrin expression has been shown to reduce LPA production due to the fact that extracellular ATX interacts with cancer cells on the plasma membrane by binding with integrins [85]. Our group found that the knockdown of CRT decreases β1 integrin expression, which we suspect may be related to ARE regulation. Notably, an ARE was also found in the 3′-UTR of β1 integrin. These data raise a novel hypothesis that ATX/LPA/VEGF-C-induced CRT may regulate β1 integrin expression via binding to the 3′-UTR of β1 integrin. Further studies will be required to elucidate the role of CRT in ARE binding and mRNA stability of VEGF-C. Furthermore, the developing understanding of mechanisms connecting LPA and hyperglycemia suggest that the combination of anti-hyperglycemic and anti-lymphangiogenic therapies may be a useful new strategy for preventing lymphangiogenesis and lymphatic metastasis in PCa.

## 6. Current Therapeutic Strategies for PCa Treatment

Current therapeutic strategies of advanced PCa, including hormone therapy, radiation therapy, and a combination of both, are available in the clinic, whereas several potential strategies have been investigated and tested in clinical trials. Receptor tyrosine kinase (RTK) inhibitors, such as cabozantinib and sitravatinib (MGCD516), have been investigated and have shown potential antineoplastic activity in advanced PCa (NCT03170960 and NCT02219711). These RTL inhibitors inhibit a closely related spectrum of RTKs including MET, AXL, MER, and members of the VEGFR, PDGFR, DDR2, TRK, and Eph families. In addition, the pan-VEGFR/Tie2 tyrosine kinase inhibitor, CEP-11981, has been investigated in regard to its potential antiangiogenic and antineoplastic activities in the phase II trial study (NCT03456804). CEP-11981 selectively binds to VEGFR and Tie2 receptor tyrosine kinases, which may result in the inhibition of endothelial cell migration, proliferation, and survival as well as the inhibition of tumor cell proliferation and tumor cell death. Interestingly, some of the downstream targets of LPA receptors also have inhibitors that are currently being tested in the clinic for advanced PCa treatment. Capivasertib and ipatasertib (GDC-0068) are orally available inhibitors of the serine/threonine protein kinase Akt that have potential antineoplastic activity. They bind to and inhibit the activity of Akt, which may result in the inhibition of the phosphatidylinositol 3-kinase (PI3K)/Akt signaling pathway and tumor cell proliferation and the induction of tumor cell apoptosis. Currently, the efficacy of capivasertib or ipatasertib combined with anti-hormone therapy is being tested in metastatic castrate-resistant prostate cancer (mCRPC) (NCT03310541 and NCT03072238). In addition, the Akt/ERK inhibitor ONC201 is also under clinical trial to treat patients with advanced solid tumors (NCT02250781). It binds to and inhibits the activity of Akt and ERK, which may result in the inhibition of the PI3K/Akt signal transduction pathway as well as the mitogen-activated protein kinase (MAPK)/ERK-mediated pathway. A phase II trial study investigated how well the MAPK/MEK/ERK inhibitor, trametinib, works in treating patients with castration-resistant PCa (NCT02881242). Trametinib is an orally bioavailable inhibitor that specifically binds to and inhibits MEK 1 and 2, resulting in the inhibition of growth factor-mediated cell signaling and cellular proliferation in various cancers. These studies will provide new insight into potential therapeutic strategies for advanced PCa.

## 7. LPA Antagonists for PCa Treatment

Since the expression and function of LPA receptors are critical for PCa progression and metastasis, selective antagonists may represent a potential treatment that can slow or prevent tumor development. Ki16425 is a selective antagonist for LPA_1_ and LPA_3_ [86], and its R-stereoisomer, Debio 0719, has been shown to inhibit bone and lung metastasis, but not tumor growth or angiogenesis in an orthotopic mouse model of breast cancer [40]. Similar results were observed in a PC-3 xenograft model of PCa, where Ki16425 treatment did not restrict the tumor blood capillary density or tumor size [20,58]. Surprisingly, however, intraperitoneal administration of Ki16425 significantly suppressed lymphangiogenesis and lymphatic metastasis in the PC-3 xenograft mouse model [20,58]. This result was the first to demonstrate that an LPA antagonist has the selective capacity to attenuate tumor lymphangiogenesis but not angiogenesis. This phenomenon might be attributable to the inhibition of LPA-induced VEGF-C expression; however, it is also possible that Ki16425 initiates differential responses in blood and lymphatic endothelial cells because the level of LPA receptor is different in the two cell types. Additional investigations are required to clarify the mechanisms that drive angiogenesis and lymphangiogenesis responses to LPA antagonists in PCa. It should be noted that no therapeutic agents targeting LPA are currently being applied in PCa patients [87]. All of the LPA agonist and antagonists are currently in the preclinical trial stage (phase I or II stage). For example, BMS-986202/AM152 (phase I) and BMS-986020 (phase II) target LPA_1_ for idiopathic pulmonary fibrosis. Preclinical studies demonstrated that Ki16425 and its R-stereoisomer Debio 0719 target LPA_1/3_ in cancers. Moreover, since the VEGFR axis has been designed and extensively tested clinically to reduce angiogenesis in advanced PCa and all the clinical trials to date have been unsuccessful, this discussion has provided new perspectives on the challenges involved in targeting lymphangiogenesis/lymphatic metastasis and VEGF-C signaling via LPA signaling in PCa.

## 8. Conclusions

**Figure 3:**
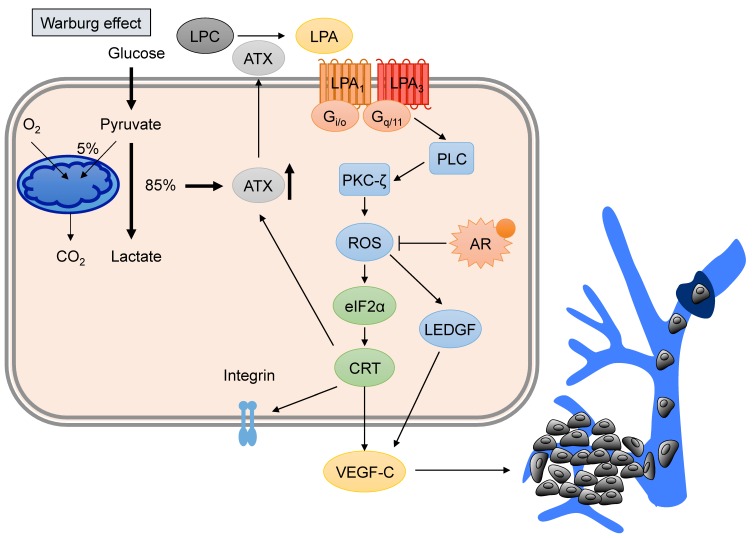
LPA–VEGF-C signaling in prostate cancer (PCa). Advanced PCa tumor cells utilize aerobic glycolysis (called the Warburg effect) to produce LPA via the promotion of autotaxin (ATX) expression. The activation of LPA signaling via LPA receptors (LPA_1_ and LPA_3_) stimulates downstream reactive oxygen species (ROS) and the ER regulators, eukaryotic initiation factor α (eIF2α) and calreticulin (CRT). Phospholipase C (PLC) and protein kinase C-ζ (PKC-ζ) are also involved in ROS production, whereas activation of the androgen receptor (AR) suppresses LPA-mediated ROS. ROS production initiates VEGF-C production and release, stimulating tumor lymphangiogenesis and lymphatic metastasis. Additionally, CRT affects the amount of membrane integrin to control cell migration. LPA promotes CRT expression, which triggers feedback regulation of ATX expression. LPC, lysophosphatidic choline. LEDGF, lens epithelium-derived growth factor.

Clinical evidence indicates that PCa often relapses as aggressive androgen-insensitive tumors after traditional hormone therapy. However, the underlying mechanisms by which androgen-dependent PCa cells transform into aggressive, metastatic androgen-insensitive tumors are still elusive. In this review, we summarized the current mechanistic knowledge of how active LPA signaling promotes VEGF-C expression, contributing to lymphangiogenesis and the progression of PCa (Figure 3). The expression profiles of LPA receptors are diverse among PCa cell lines, with more malignant cell lines exhibiting LPA receptor-mediated effects in PCa progression. In addition, ATX, the key enzyme for extracellular biosynthesis of LPA, is highly expressed in advanced PCa cells, and an LPA-CRT-ATX positive feedback loop promotes lymphangiogenesis and metastasis in PCa. Moreover, VEGF-C mediates LPA-induced lymphangiogenesis in PCa, and hyperglycemia was also found to play a role in promoting LPA/VEGF-C production, contributing to tumorigenesis in advanced PCa. Taken together, these lines of evidence indicate that LPA signaling plays a crucial role in PCa progression. Thus, further investigations into LPA production, LPA receptor expression, and VEGF-C-induced lymphangiogenesis will provide valuable knowledge regarding the molecular mechanisms of PCa progression. Targeting these mechanisms that comprise the LPA–VEGF–C axis offers a new therapeutic strategy for PCa.
